# CD151-α3β1 integrin complexes are prognostic markers of glioblastoma and cooperate with EGFR to drive tumor cell motility and invasion

**DOI:** 10.18632/oncotarget.4896

**Published:** 2015-08-13

**Authors:** Pengcheng Zhou, Sonia Erfani, Zeyi Liu, Changhe Jia, Yecang Chen, Bingwei Xu, Xinyu Deng, Jose E. Alfáro, Li Chen, Dana Napier, Michael Lu, Jian-An Huang, Chunming Liu, Olivier Thibault, Rosalind Segal, Binhua P. Zhou, Natasha Kyprianou, Craig Horbinski, Xiuwei H. Yang

**Affiliations:** ^1^ Department of Cancer Biology and Pediatric Oncology, Dana-Farber Cancer Institute and Harvard Medical School, Boston, MA, USA; ^2^ Department of Pharmacology and Nutritional Sciences, Markey Cancer Center and University of Kentucky, Lexington, KY, USA; ^3^ Department of Respiratory Medicine, First Affiliated Hospital of Soochow University, Suzhou, Jiangsu Province, P. R. China; ^4^ Department of Gastroenterology, Provincial People's Hospital, Zhengzhou, Henan Province, P. R. China; ^5^ Department of Chemistry, University of Kentucky, Lexington, KY, USA; ^6^ Department of Pathology and Laboratory Medicine, Markey Cancer Center and University of Kentucky, Lexington, KY, USA; ^7^ Department of Molecular and Cellular Biochemistry, Markey Cancer Center and University of Kentucky, Lexington, KY, USA; ^8^ Department of Biomedical Science, Florida Atlantic University, Boca Raton, FL, USA; ^9^ Department of Urology, Markey Cancer Center and University of Kentucky, Lexington, KY, USA

**Keywords:** glioblastoma, CD151, α3 integrin, EGFR, cell invasion and motility

## Abstract

Glioblastoma, one of the most aggressive forms of brain cancer, is featured by high tumor cell motility and invasiveness, which not only fuel tumor infiltration, but also enable escape from surgical or other clinical interventions. Thus, better understanding of how these malignant traits are controlled will be key to the discovery of novel biomarkers and therapies against this deadly disease. Tetraspanin CD151 and its associated α3β1 integrin have been implicated in facilitating tumor progression across multiple cancer types. How these adhesion molecules are involved in the progression of glioblastoma, however, remains largely unclear. Here, we examined an in-house tissue microarray-based cohort of 96 patient biopsies and TCGA dataset to evaluate the clinical significance of CD151 and α3β1 integrin. Functional and signaling analyses were also conducted to understand how these molecules promote the aggressiveness of glioblastoma at molecular and cellular levels. Results from our analyses showed that CD151 and α3 integrin were significantly elevated in glioblastomas at both protein and mRNA levels, and exhibited strong inverse correlation with patient survival (*p* < 0.006). These adhesion molecules also formed tight protein complexes and synergized with EGF/EGFR to accelerate tumor cell motility and invasion. Furthermore, disruption of such complexes enhanced the survival of tumor-bearing mice in a xenograft model, and impaired activation of FAK and small GTPases. Also, knockdown- or pharmacological agent-based attenuation of EGFR, FAK or Graf (ARHGAP26)/small GTPase-mediated pathways markedly mitigated the aggressiveness of glioblastoma cells. Collectively, our findings provide clinical, molecular and cellular evidence of CD151-α3β1 integrin complexes as promising prognostic biomarkers and therapeutic targets for glioblastoma.

## INTRODUCTION

Glioblastomas, grade IV gliomas by World Health Organization (WHO) classification, belong to one of the most aggressive types of brain cancer [1]. Current therapeutic management of glioblastomas involves surgical resection and adjuvant chemoradiation. Despite advances made in imaging and treatment modalities, the prognosis of glioblastoma remains very dismal with five-year patient survival as low as 3% [1]. This fatal type of brain cancer is characterized by tumor cells with highly motile and invasive capacities [2]. As such, glioblastoma is considered an infiltrating disease with a strong tendency to escape surgical or other therapeutic treatments [1].

The progression and recurrence of glioblastoma has been strongly linked to the activation of receptor tyrosine kinases (RTKs) and their signaling pathways [3]. In particular, the majority of glioblastomas exhibit activated signaling of epidermal growth factor receptor (EGFR), which occurs through gene amplification, activating mutations, and/or receptor overexpression [4]. Aside from promoting cell proliferation, EGFR signaling drives the motility and invasiveness of glioblastoma cells. Unfortunately, the clinical efficacy of EGFR-specific inhibitors in glioblastoma have fallen below expectations [5].

There is growing evidence that the malignancy of glioblastoma is strongly promoted by integrins, a family of heterodimeric receptors. This class of adhesion molecules supports cell-extracellular matrix (ECM) interactions, thereby permitting tumor cell migration and invasion [6]. As members of the integrin family, the laminin-binding (LB) integrins, including α3β1, α6β1 and α6β4, are highly expressed in cultured glioblastoma cells [7–9] or a subset of CD133^+^ glioma stem cells [10]. They are also implicated in promoting the migration and invasion of glioblastoma cells [7, 11]. In contrast, several RGD-binding integrins, including α5β1 and αvβ3 integrins, appear to promote tumor cell survival and/or drug resistance of glioblastomas [12, 13]. These observations raise the likelihood that integrins, particularly LB integrins, are candidate regulators of glioblastoma malignancy, and may serve as biomarkers and/or therapeutic targets for this aggressive disease.

The pro-malignant function and signaling of LB integrins are coordinated by members of the tetraspanin family, including CD151, CD9, CD81, and CD82 [14–16]. Notably, tetraspanins form tight protein complexes with LB integrins on the cell surface through protein-protein or protein-lipid interactions [17–19]. However, only CD151 directly interacts with α subunits of LB integrins at their extracellular portions, in which it dictates integrin activation and signaling by influencing their lateral clustering on the cell surface [18, 20]. It is of no surprise that CD151 is frequently implicated in promoting tumor progression by enhancing either α3 or α6 integrin-dependent cellular processes [11, 21, 22]. There is also evidence that CD151 mediates LB integrin-dependent activation of multiple signaling pathways [11]. In addition, there are reports of links between CD151 expression and tumor grade or patient survival for several cancer types. Together, these available observations implicate that, like its associated LB integrins, CD151 is a potential key player in the progression of glioblastoma. How CD151 and its associated LB integrins are associated with the aggressiveness of glioblastoma, however, still remains largely unclear, particularly at the clinical, cellular, and signaling levels.

Here, we report clinical and functional analyses of CD151 and its associated α3 integrin in glioblastoma. We applied annotated tissue microarrays (TMAs) to evaluate the correlation between the expression of CD151 or α3β1 integrin and clinicopathological parameters, including tumor grade, *IDH1* mutation status, and patient survival. Additionally, we performed functional studies of multiple glioma cell lines to assess the impact of CD151 ablation on glioma aggressiveness, particularly regarding cell motility and invasiveness. Finally, signaling analyses were conducted to identify key effectors downstream of CD151-LB integrin complexes. Results from these analyses demonstrate that CD151 and α3β1 integrin are key drivers of glioblastoma aggressiveness, and serve as independent prognostic markers and promising therapeutic targets.

## RESULTS

### Clinical association between CD151 and glioma malignancy

**Figure 1:**
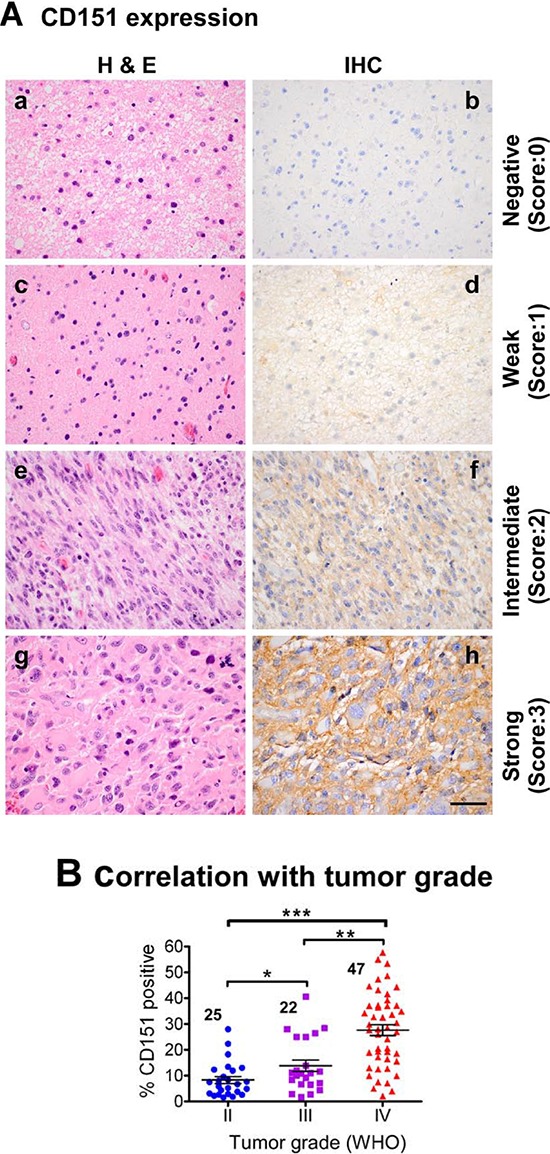

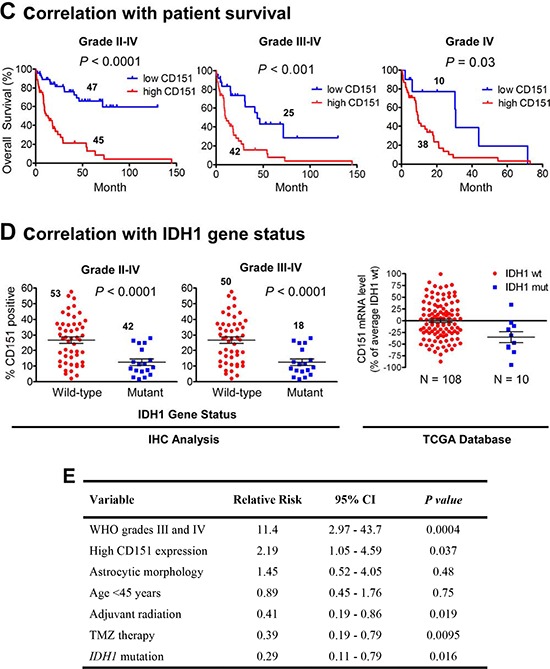
Relationship between CD151, WHO tumor grade, patient survival, and IDH1 gene status in a TMA-based glioma patient cohort TMAs harboring 96 patient glioma tissues were subjected to H&E staining and IHC analyses of the CD151 protein. **A.** Representative images of H&E staining (a, c, e & g) and corresponding CD151 antibody staining (b, d, f & h) of glioma tissues. **B.** CD151-positive staining (by percentage) versus tumor grade. *P* values indicated, *: *p* < 0.05; **: *p* < 0.01; ***: *p* < 0.001. **C.** Correlation between CD151 expression and overall patient survival (OS); *, *p* < 0.05. Data shown for analyses of patient cohorts consisting of (a) WHO grade II-IV gliomas or (b) grade III-IV gliomas or (c) only IV gliomas, i.e., glioblastoma. **D.** Correlation between CD151 and status of IDH1 gene from our TMA-based patient cohort (*left and middle panels*) or TCGA glioblastoma samples (*right panel*). For B-D, an in-house TMA-based cohort was stratified into CD151-low and high groups based on IHC staining of CD151 and corresponding sample sizes indicated. The correlation between CD151 expression and patient survival was evaluated by the Log-rank test. **E.** Multi-variant analyses of CD151 as an independent prognostic factor of gliomas. Independent prognostic factors were determined by Cox proportional hazards analysis of an in-house TMA-based patient cohort (*n* = 88). TMZ, temozolomide. CI, confidence interval. Scale bar: 50 μm.

To evaluate the clinical significance of CD151 in glioma malignancy, we carried out immunohistochemistry (IHC) analyses with a TMA containing 96 paraffin-embedded patient glioma tissues. As shown in Fig. 1A, CD151 staining was primarily localized on the plasma membrane of tumor cells and detectable in the cytoplasm. The number of CD151-positive tumors in the glioblastoma group, that is, WHO grade IV gliomas, was more than two-fold higher than their low-grade counterparts (Fig. 1B). To evaluate the clinical significance of aberrant CD151 expression, the patient cohort was divided into CD151-low (<15% cells positive) and CD151–high groups (≥15% cells positive), as determined by Cutoff Finder (http://molpath.charite.de/cutoff/index.jsp) [23]. Our data showed that patients belonging to the CD151-high group had poorer survival than their counterparts, regardless of how patient samples were pooled by tumor grade (Fig. 1C). A similar trend was also detected from our analyses of a commercial glioma TMA (data not shown).

Because *IDH1* gene status is a powerful prognostic indicator for infiltrative gliomas [24], we also evaluated the relationship between its mutation status and CD151 expression in our patient cohort. As shown in Fig. 1D, CD151 protein was significantly lower in gliomas with mutant *IDH1*. A similar trend was detected for CD151 mRNA in The Cancer Genome Atlas (TCGA) glioblastoma dataset. Importantly, a multivariate analysis of our patient cohort showed that elevated CD151 expression was a better independent adverse prognostic marker, compared to patient age, IDH1 status or the treatment with temozolomide, a standard chemotherapeutic agent (Fig. 1E). Together, these data demonstrate a strong association between CD151 expression and glioma aggressiveness.

### CD151 promotes glioma cell motility and invasion

**Figure 2:**
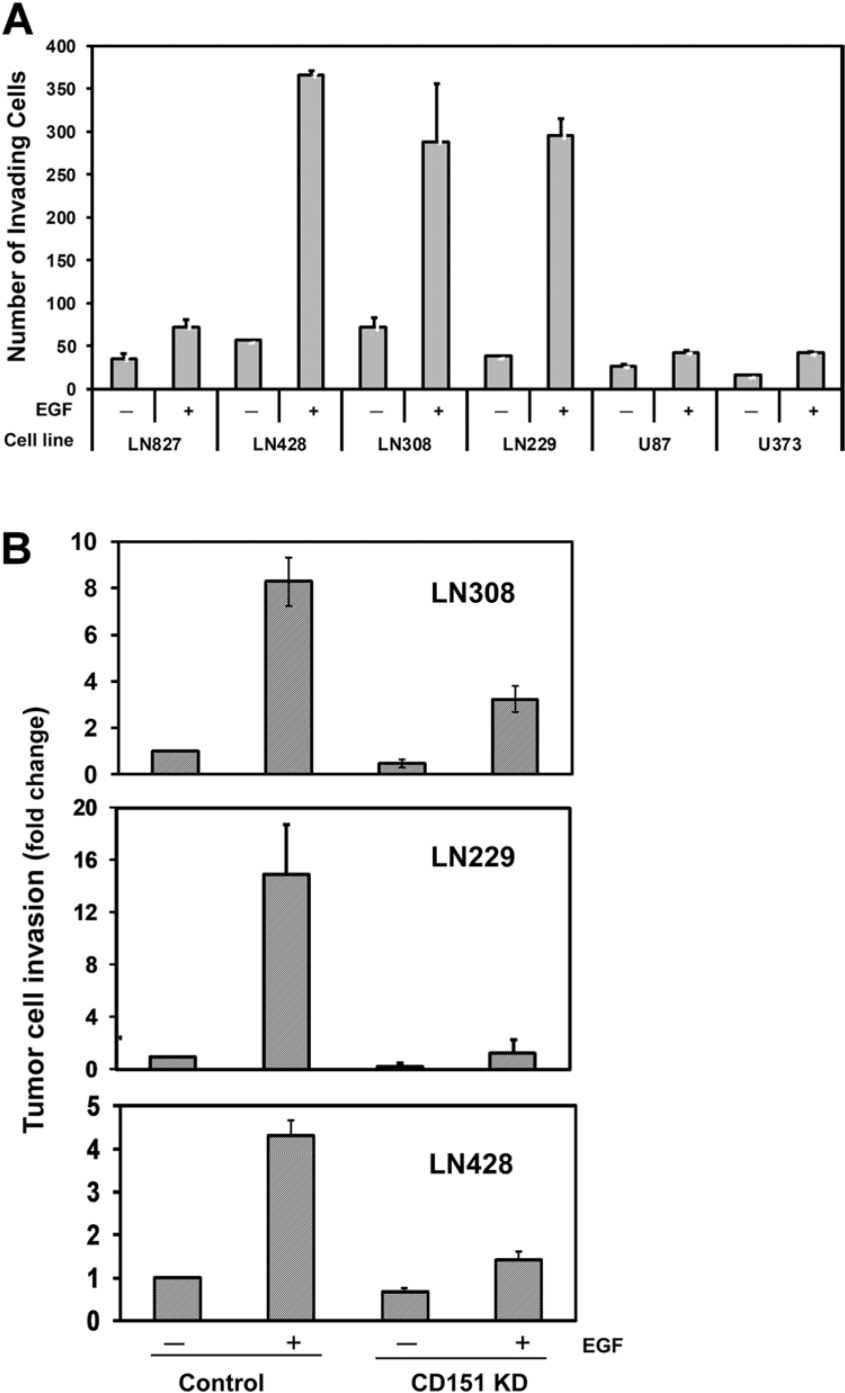

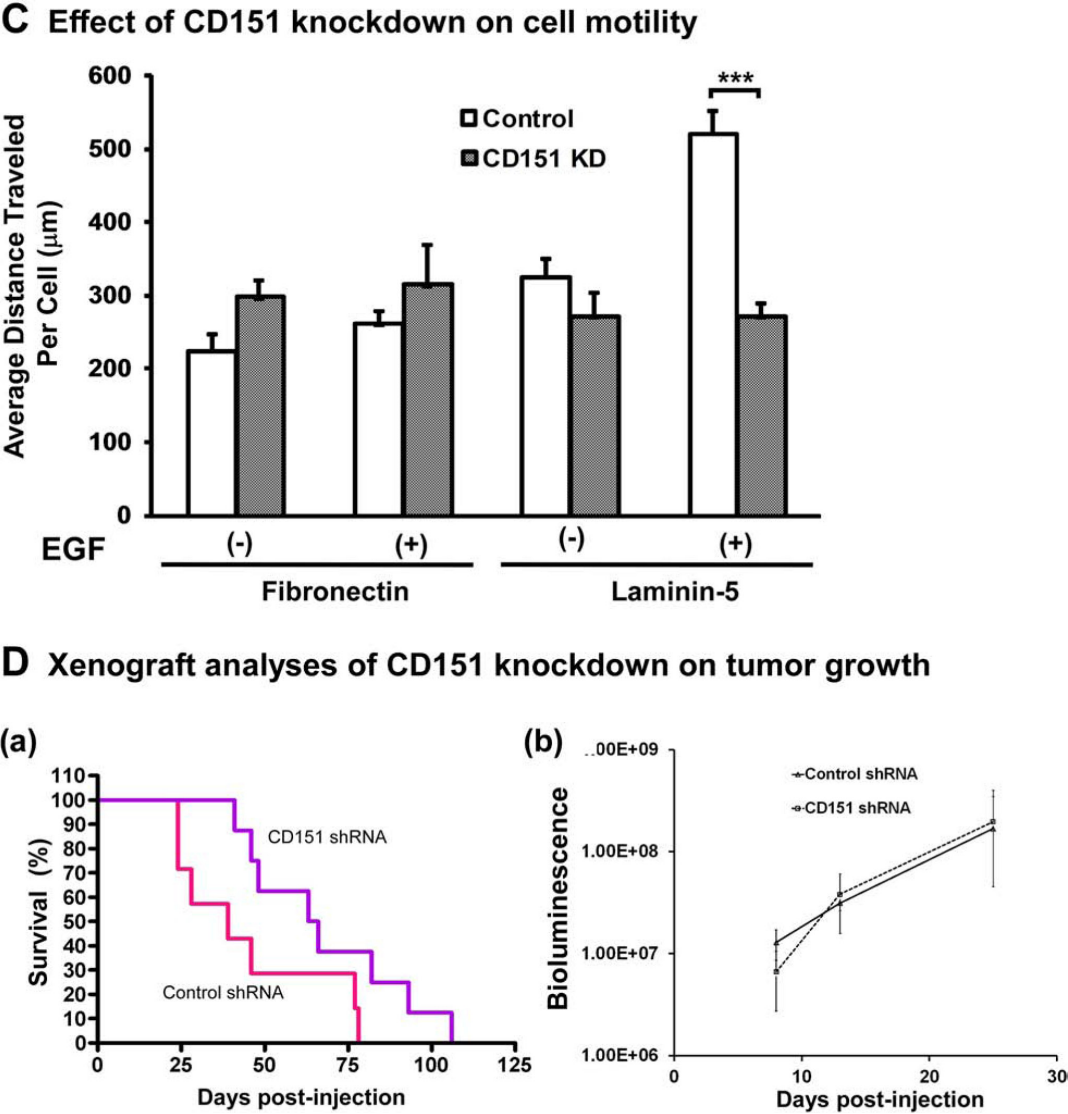
A role of CD151 in EGF-induced glioblastoma cell motility, invasion, and *ex vivo* tumor formation **A.** EGF-stimulated invasiveness of cultured glioblastoma cell lines. Glioblastoma cell lines including LN827, LN428, LN308, LN229, U87 and LN373 were subjected to an invasion assay with respect to the presence and absence of EGF. 1.0 × 10^5^ tumor cells were placed on the top of a Matrigel-coated insert in a 24-well plate and allowed to invade for 17–20 hrs before being fixed. Number of invaded cells is shown (mean ± SEM, *n* = 3). **B.** Effect of CD151 knockdown (KD) on the invasion of LN308 and LN229 cells (fold change ± SEM, *n* = 3). The efficiency of stable knockdown of CD151 was assessed by FACS analyses (see Supplementary Fig. S2). **C.** Effect of CD151 KD on glioma cell motility. Random cell motility was measured for LN299 cells with or without CD151 KD. The analyses were conducted in triplicate and average distance traveled by individual cells were estimated. A total of 25–35 individual cells per treatment were tracked. For A-C, EGF: 10 ng/ml. **D.** Xenograft analyses of CD151 ablation on tumor growth. Mice injected with control or CD151 knockdown LN229 cells at 10 animals per group monitored over the indicated periods of times. *(a)* Survival curve of tumor-bearing mice. The *p*-value was obtained by Log-rank test; *(b)* Change in the bioluminescence of injected mice.

Next, we conducted *in vitro* analyses to test if CD151 functionally contributed to the aggressiveness of gliomas as suggested by our clinical analyses (Fig. 1). Our FACS analyses indicated that CD151 and LB integrins were highly expressed across a panel of glioblastoma cell lines (Supplementary Fig. S1). The strong expression of other tetraspanins, including CD9 and CD81, was also detected, consistent with a recent report [25]. According to our Matrigel-based invasion assay, these tumor cell lines exhibited a wide range of variation in invasiveness (Fig. 2A). In particular, the invasive capabilities of LN428, LN308 and LN229 lines increased by 3.5- to 5-fold upon EGF stimulation, consistent with the strong pro-malignant function of EGFR in glioblastomas [26, 27]. Because CD151 or its associated LB integrins have been shown to functionally collaborate with EGFR in other cancer types [15, 28], these highly EGF-responsive cell lines were adopted for subsequent functional analyses.

In the three cell lines most sensitive to EGF (LN229, LN308, and LN428), CD151 knockdown led to a 2- to 7-fold decrease in the number of tumor cells invading through the Matrigel after EGF stimulation (Fig. 2B). Also, EGF-stimulated random cell motility was mostly abolished upon CD151 ablation when LN229 cells were engaged with laminin-5, i.e., laminin 332, but not fibronectin (Fig. 2C). However, CD151 knockdown had only minimal effect on the proliferation of glioma cells (data not shown). In line with these *in vitro* observations, CD151 knockdown extended the survival of tumor-bearing mice with orthotopic LN229 glioblastoma xenografts (Fig. 2D, *p* < 0.05), even though minimal change occurred in tumor growth. These *in vitro* and *ex vivo* observations demonstrate that CD151 promotes glioblastoma progression by promoting tumor cell motility and invasiveness, rather than impacting cell proliferation or tumor growth.

### Functional and clinical links of CD151-associated α3β1 integrin to glioblastoma malignancy

**Figure 3:**
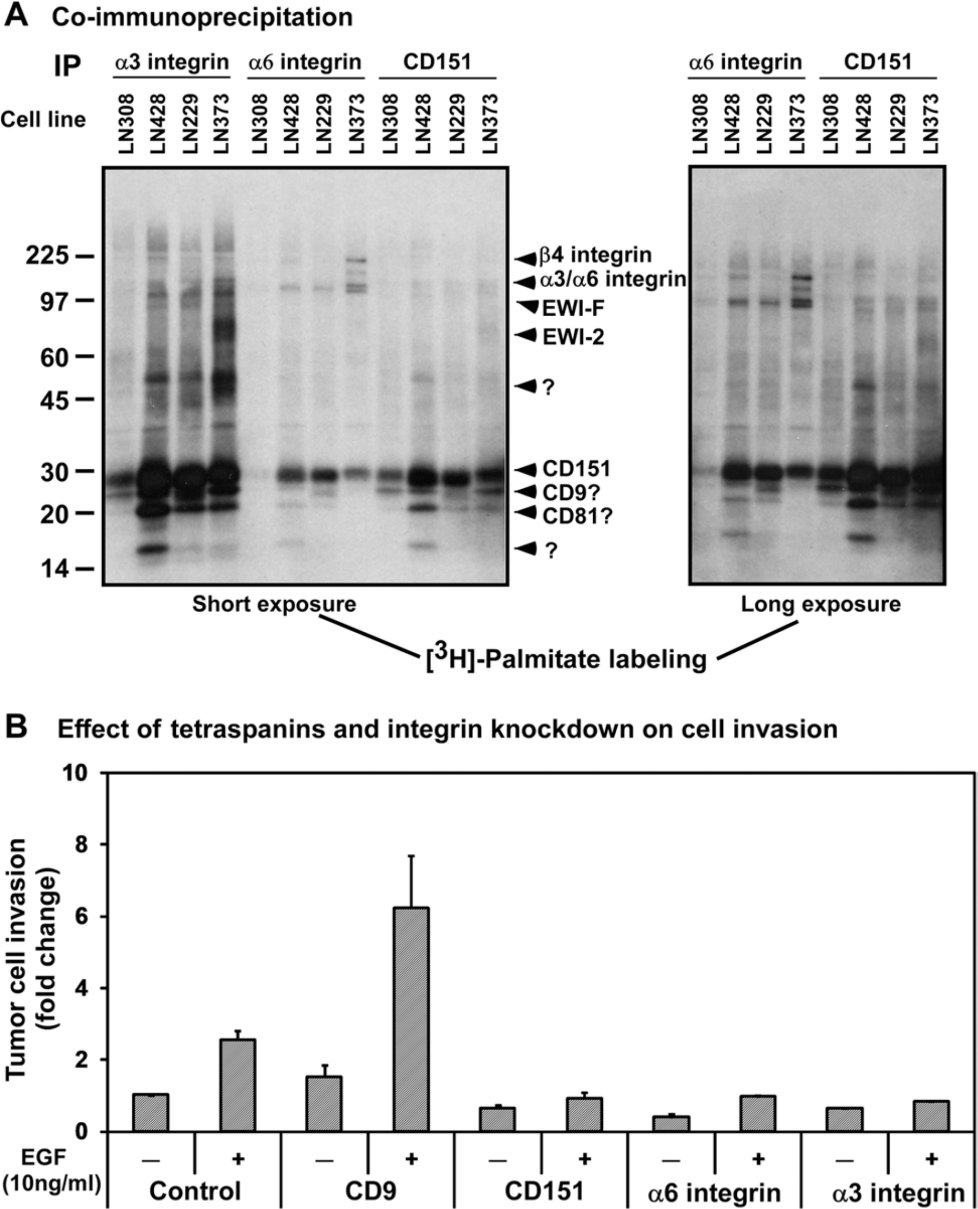

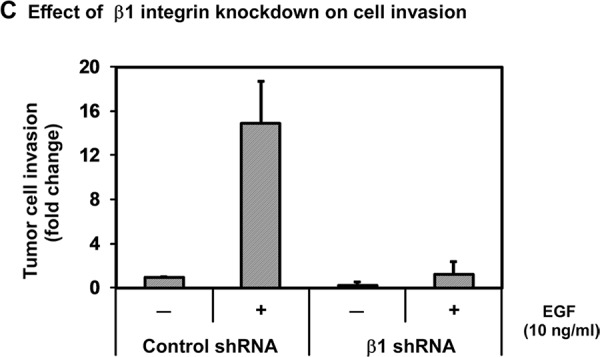
The pro-invasive function of CD151-associated protein complexes in glioblastoma cells **A.** Co-immunoprecipitation of CD151 and LB integrins. Lysates of [^3^H]-palmitate-labeled glioma tumor cells were subjected to immunoprecipitation with the indicated antibodies, followed by radiographic detection. *Left panel*, short exposure; *right panel*, long exposure. **B.** Impact of disrupting CD151, and its associated integrins and tetraspanin molecules on the invasion of glioma cells. LN229 cells were treated with scramble (control) or siRNA against CD9, CD151, α3 and α6 integrins. Fold changes were calculated relative to the number of invaded cells in the group treated with control siRNA in the absence of EGF stimulation. **C.** Effect of stable knockdown of β1 integrin on the invasion of LN229 cells. EGF: 10 ng/ml. B-C The efficiency of tetraspanin or integrin knockdown was estimated by immunoblotting or FACS analysis (see Supplementary Fig. S3, panels A-B).

Tetraspanin CD151 is implicated in mediating diverse physiological and pathological processes by impacting the function or signaling of its associated protein complexes, instead of directly interacting with signaling molecules [17, 28]. Such complexes are also readily detected by the abilities of integrins and tetraspanin molecules to incorporate palmitate and form tetraspanin-enriched membrane microdomains (TEMs) [17]. Therefore, we conducted [^3^H]-palmitate labeling and co-immunoprecipitation analyses of multiple glioblastoma cell lines under 1.0% Brij96 condition. Our data showed that CD151 was present in the protein complexes of α3 and α6 integrins in multiple glioma cell lines (Fig. 3A). In addition, several other tetraspanins, including CD9, CD81 and CD82, appeared to exist in the complexes. The profile of these labeled proteins was also consistent with our prior studies [16, 29], and supported by the surface expression of these molecules from our FACS analyses (Supplementary Fig. S1).

Functional analyses were subsequently performed to evaluate the contribution of such protein complexes to the aggressiveness of gliomas. As shown in Fig. 3B, knockdown of α3 or α6 integrin was as effective as CD151 ablation in inhibiting EGF-induced invasion of LN229 cells. In contrast, CD9 knockdown enhanced both basal and EGF-induced invasion of glioma cells, consistent with its anti-invasive role as shown in prior studies [25, 28]. In further support of these observations, we found that the stable knockdown of β1 integrin led to a marked inhibition in the invasion of LN229 cells (Fig. 3C). Together, these data illustrate that CD151 and its associated LB integrins form protein complexes and cooperate to promote the invasiveness of glioblastomas.

**Figure 4:**
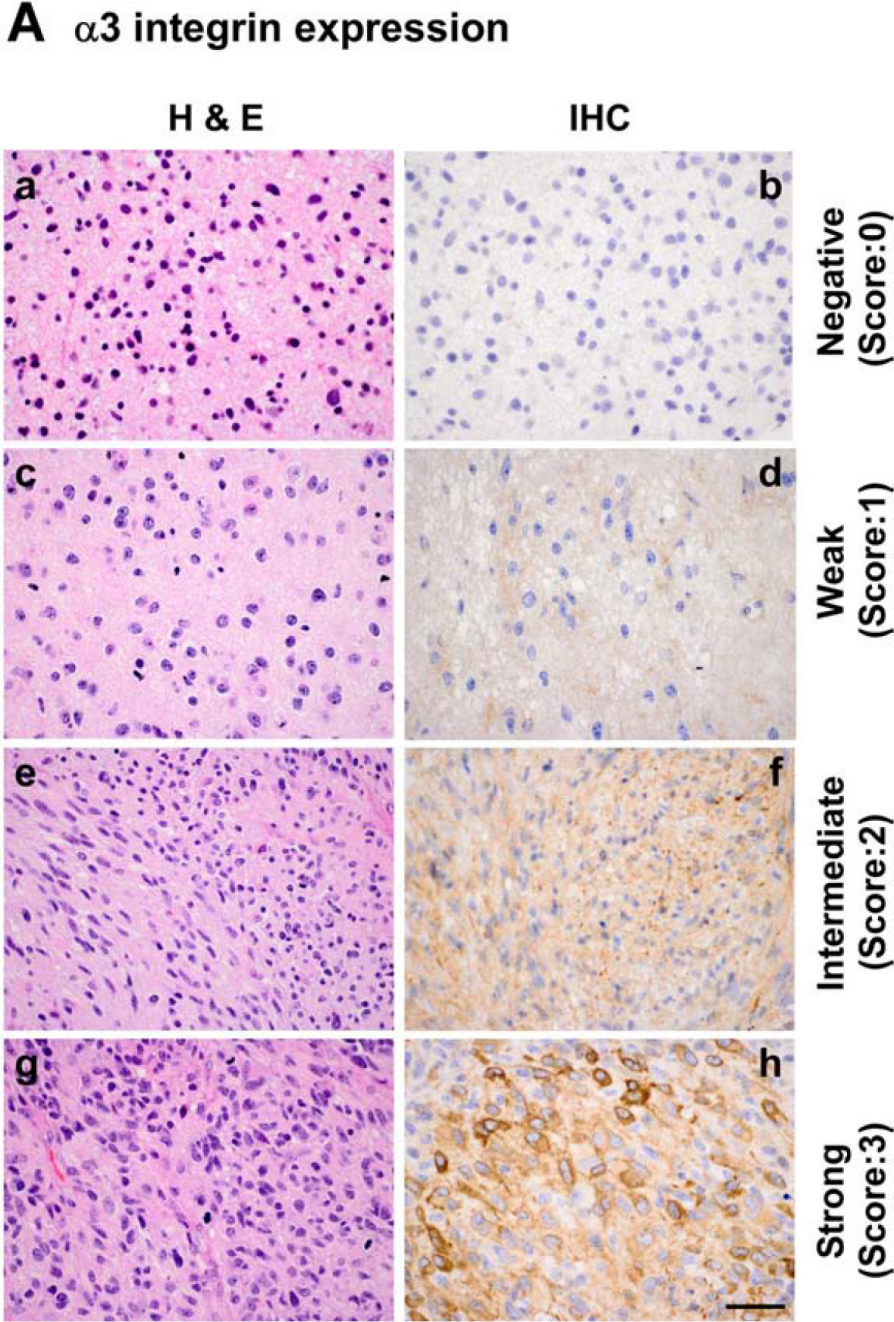

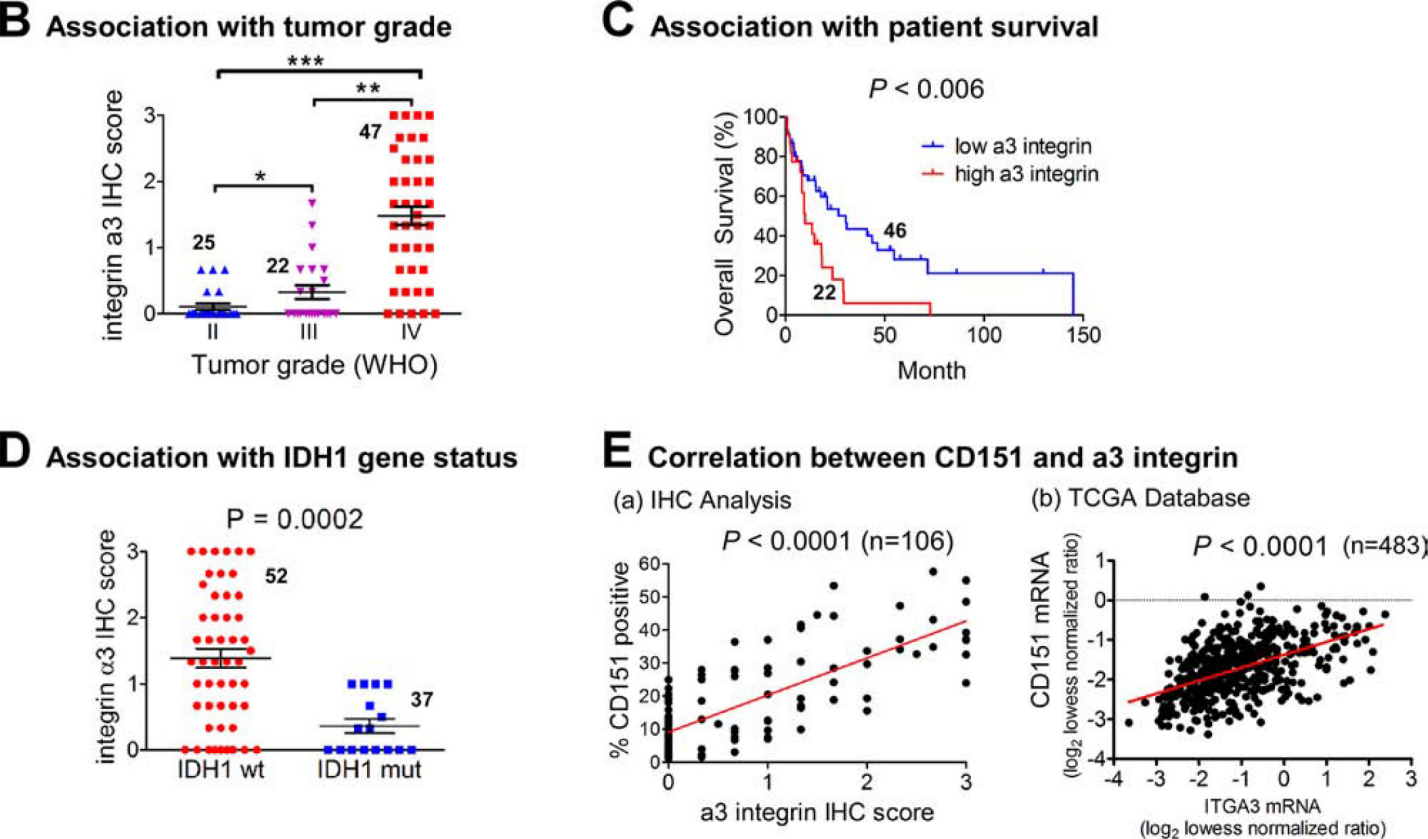
Relationship between α3 integrin, WHO tumor grade, IDH1 gene status, patient survival and CD151 expression in a TMA-based glioma patient cohort and TCGA glioblastomas **A.** Representative images of H&E (a, c, e, g) and IHC staining (b, d, f, h) of an in-house glioma TMA as described in Figure 1. IHC analyses were conducted with a polyclonal antibody against α3 integrin. a-b, grade II; c-d grade II; e-f, grade III; g-h, grade IV or glioblastoma. *P* values indicated. **B.** A plot of percentages of α3 integrin-positive staining versus tumor grade. *P* values are indicated (*p < 0.05; **p < 0.01; ***p < 0.001). **C.** Association of α3 integrin and overall patient survival (OS) in WHO grade III-IV gliomas. The patient cohort was divided into low and high α3 integrin expression groups. The cutoff was at ≥ 3 IHC staining of α3 integrin. The significance of the correlation between α3 integrin and patient's survival was assessed by the Log-rank test. **D.** The association between CD151 expression and status of IDH1 gene from IHC or TCGA database analyses. **E.** Correlation between CD151 and α3 integrin expression in the TMA cohort of grade II-IV gliomas at protein level (*left panel*) or at mRNA level (*right panel*). The mRNA expression of these genes was obtained in the external TCGA glioblastomas dataset. Sample sizes indicated. Scale bar: 50 μm.

Next, analyses of clinical samples were conducted to evaluate the link of CD151-associated α3 integrin to the malignancy of gliomas. As shown in Fig. 4A–B, expression of α3 integrin was detectable in plasma membrane and cytoplasmic compartments of tumor cells, and was markedly elevated in high-grade gliomas including glioblastomas. Again, by using 15% cells being α3 integrin-positive as a cutoff value, we found that elevated expression of α3 integrin was strongly correlated with poor patient survival (*p* < 0.006) (Fig. 4C). There was also a significant negative correlation between the level of this integrin and the mutation status of *IDH1* (Fig. 4D). Protein expression of CD151 and α3 integrin were strongly correlated in our patient cohort (slope = 11.2 ± 0.93, R^2^ = 0.58, *p* < 0.0001) (Fig. 4E). In addition, a similar association occurred at the mRNA level, according to our analyses of the TCGA glioma dataset (Fig. 4E). Together, our analyses of two independent clinical cohorts consistently suggest that CD151 and α3 integrin act together to promote the aggressiveness of gliomas.

### CD151-α3β1 integrin complexes drive tumor cell motility and invasion via FAK activation

**Figure 5:**
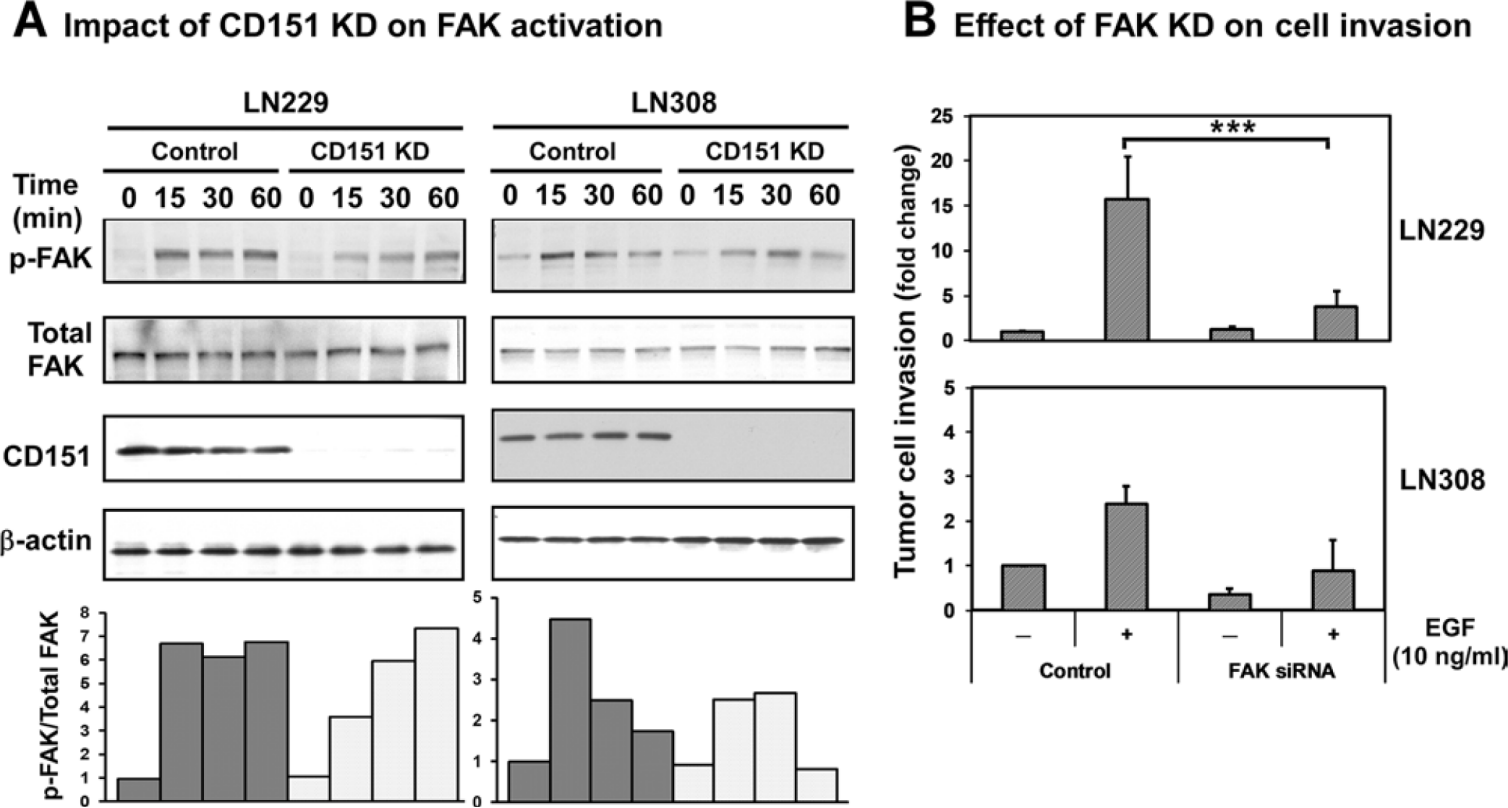

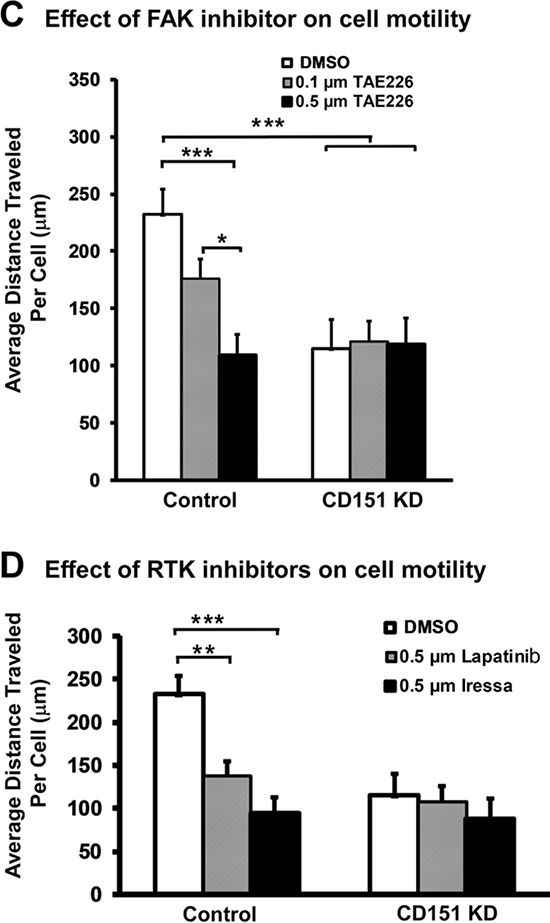
Activation of FAK in glioma cells by CD151-α3β1 integrin complexes and their crosstalk with EGFR **A.** Effect of CD151 ablation on integrin-dependent phosphorylation of FAK-Y^397^ residue. LN229 or LN308 cells with or without stable CD151 KD were transiently placed onto laminin-5 substrate for the indicated periods of time, followed by lysing in RIPA buffer and immunoblotting. *Top panel*, immunoblotting of total or phosphorylated FAK. *Bottom panel*, ratios of FAK-Y^397^/Total FAK proteins determined by densitometry. **B.** The impact of FAK knockdown on the invasion of either LN229 or LN308 glioma cells. FAK was knocked down via siRNA and confirmed by immunoblotting (see Supplementary Fig. S3, panel C). **C–D.** The impact of FAK or EGFR inhibition on EGF-mediated random tumor cell motility in control and CD151 KD LN229 cells. Inhibition of FAK and EGFR was carried out by using TAE226 or Lapatinib or Gefitinib at 0.1 μM or 0.5 μM.

Recent studies have implicated that FAK is a putative driver of glioma malignancy and acts downstream of CD151-integrin complexes [15, 28]. Hence, we examined the signaling link of FAK activation to CD151-integrin complex-mediated glioma aggressiveness. Our data showed that the stable knockdown of CD151 caused a delay or decrease in the phosphorylation of FAK-Y^397^ residue induced by the engagement of α3β1 integrin and its physiological ligand, laminin-5, during the transient spreading of either LN229 or LN308 cells (Fig. 5A). In addition, FAK knockdown reduced the EGF-induced invasion of LN229 and LN308 cells by 2.3- to 3.5-fold (Fig. 5B), respectively. In line with these observations, EGF-induced cell motility was also markedly impaired when FAK was inhibited by the use of a small molecule-based inhibitor, TAE226 [30]. Such inhibition was nearly equally effective as CD151 knockdown (Fig. 5C). Moreover, the involvement of EGFR in the motility of glioma cells was independently validated by our analyses with two clinically relevant EGFR inhibitors, lapatinib/Tykerb and gefitinib/Iressa (Fig. 5D). Taken together, these results illustrate that FAK promotes the aggressive traits of gliomas by acting downstream of CD151-integrin complexes and EGFR.

### Graf (ARHGAP26) as a key downstream effector of CD151-α3β1 integrin complex signaling

**Figure 6:**
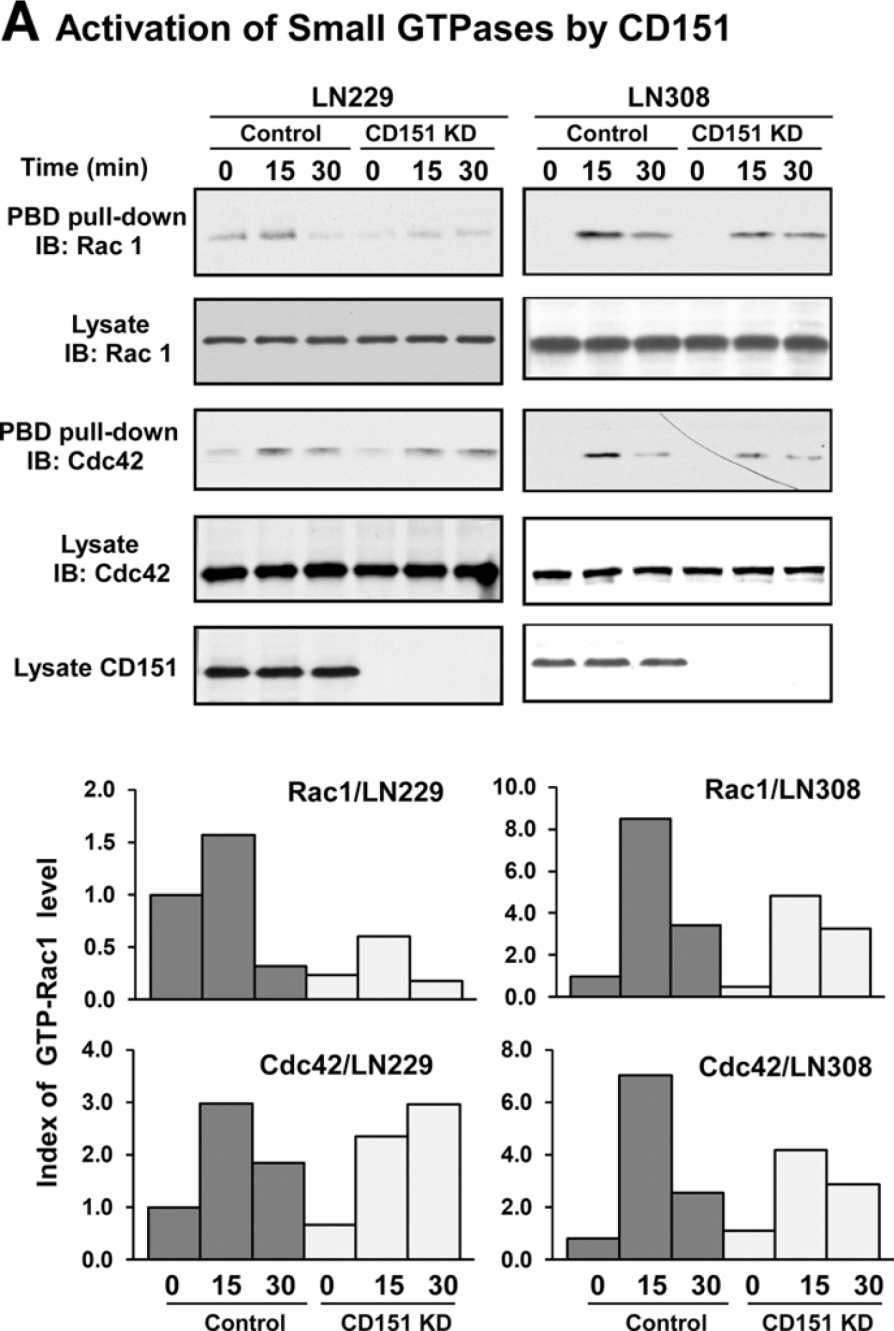

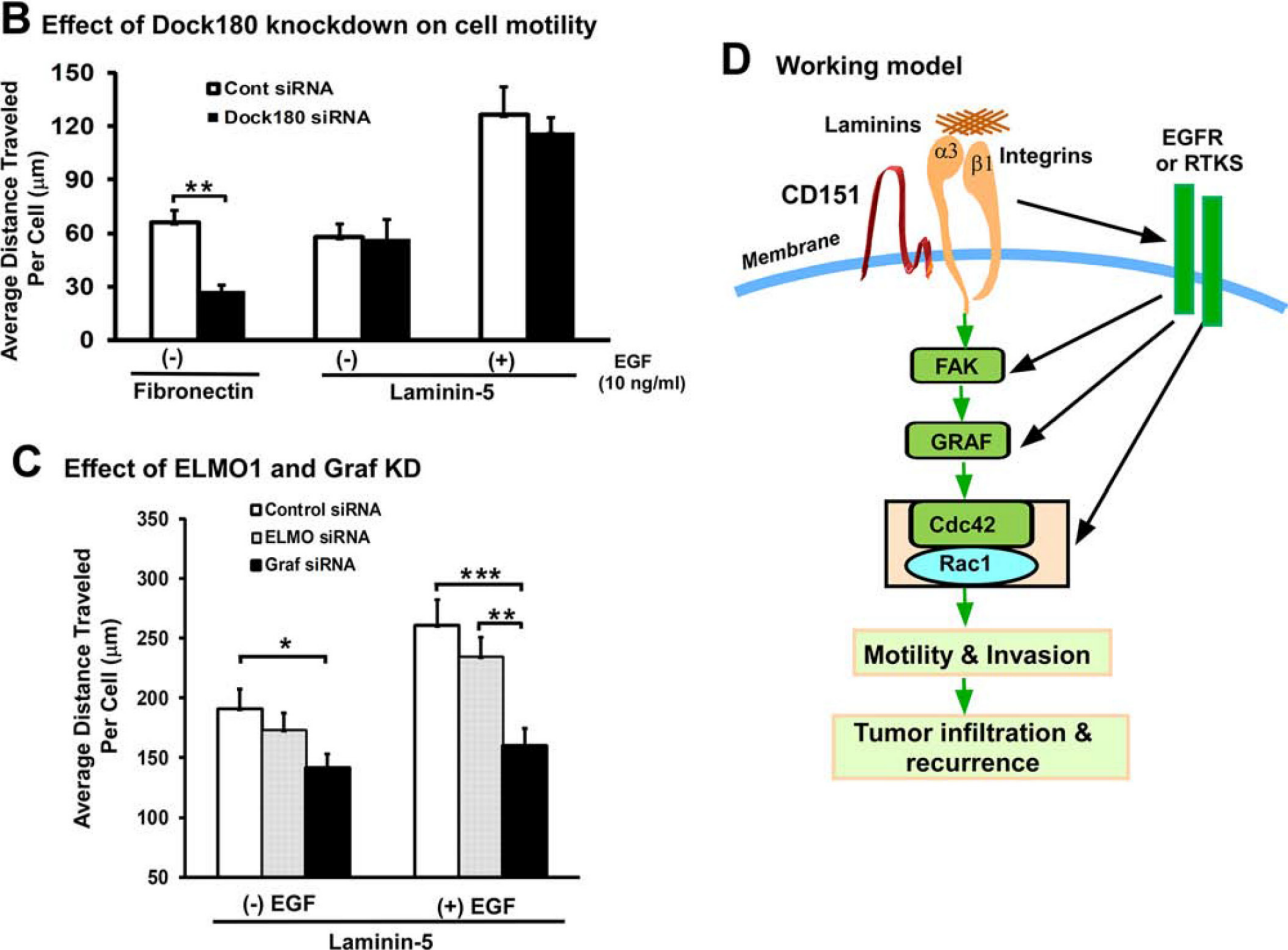
A functional link between CD151-LB integrin complexes and the GRAF/Small GTPase signaling axis **A.** Effect of CD151 ablation on integrin-dependent activation of small GTPase Rac1 and Cdc42 in LN229 and LN308 cells. Control and CD151 KD cell were detached and transiently engaged with laminin-5 substrate for the indicated periods of time, followed by lysing in RIPA buffer and immunoblotting. *Top panel*, immunoblotting of Rac1 in GTP-bound form or present in cell lysates. *Bottom panel*, indices of GTP-bound Rac1 protein determined by densitometry. **B-C.** Effect of DOCK180, ELMO1, and GRAF/ARHGAP26 knockdown on integrin-mediated motility of LN229 glioma cells. The average distance traveled by tumor cells was calculated. Values: mean ± SEM (*n* = 15). Efficiency of DOCK180, ELMO1, and GRAF/ARHGAP26 knockdown was assessed by immunoblotting (see Supplementary Figure S3, panels D-E). EGF: 10 ng/mL. **D.** A schematic illustration of a working model of CD151-α3β1 integrin complexes in glioblastoma.

Integrins have recently been implicated in promoting the motility of glioma cells via activation of multiple small GTPases including Rac1 and Cdc42 [31, 32]. Thus, we sought to determine whether a similar link existed in glioma cells. Our data showed that CD151 knockdown markedly impaired the activation of Rac1 or Cdc42 GTPases upon engagement of α3 integrin with laminin-5 in LN229 or LN308 cells (Fig. 6A). Next, we sought to identify which specific GDP/GTP exchange factors or RhoGAPs acted downstream of CD151-integrin complexes. As shown in Fig. 6B, disruption of DOCK180 impaired motility of LN229 cells on fibronectin but not laminin-5 substrate. A similar effect was found when ELMO1, another motility-related adaptor molecule strongly implicated in the aggressiveness of gliomas [33], was knocked down (Fig. 6C). However, only disruption of Graf (ARHGAP26) led to a marked decrease in the motility of LN229 cells on laminin-5, regardless of EGF stimulation (Fig. 6C). Together, these observations indicate a crucial role of the Graf (ARHGAP26)/Rac1/Cdc42 axis, not DOCK180 or ELMO1, in CD151-α3 integrin complex-mediated signaling in glioblastoma.

## DISCUSSION

Results from our studies support a close link between CD151-α3β1 integrin complexes and the aggressiveness of glioblastomas. Our analyses of TMA-based glioma patient cohort as well as TCGA glioma dataset consistently indicate that the expression of CD151 or α3β1 integrin is strongly correlated with tumor grade, *IDH1* gene status, and patient survival. Our biochemical analyses reveal the presence of CD151-α3β1 integrin complexes in glioblastoma cells. Disruption of CD151 or α3β1 integrin markedly impairs EGF/EGFR-evoked tumor cell motility and invasiveness. At the signaling level, CD151-α3β1 integrin complexes cooperate with EGFR to activate FAK- and Graf (ARHGAP26)/Rac1/Cdc42 axis-mediated pathways. Together, these findings provide strong clinical, cellular and molecular evidence that CD151-α3β1 integrin complexes and associated pathways are crucial players in the progression of glioblastoma or other malignant gliomas (Fig. 6D).

### Aberrant expression of CD151 and α3β1 integrin as independent prognostic factors

The current study provides strong clinical evidence for the close link between CD151 or its associated α3β1 integrin and the malignancy of human glioblastoma. Our TMA-based analyses reveal significant correlation between CD151 or its associated α3β1 integrin and tumor grade, patient survival, and IDH1 gene status (Figs. 1, 4). These results are also in accord with recent reports of CD151 expression in glioblastomas [34, 35]. Moreover, our data indicate a significant concordance in the expression of CD151 and α3β1 integrin in our patient cohort and TCGA gliomas (Fig. 4E). As such, our findings support CD151 and α3β1 integrin as potential biomarkers for glioblastoma.

It is of clinical significance to know how expression of CD151 or α3β1 integrin is markedly elevated in glioblastomas at both the mRNA and protein levels. Based on prior biochemical analyses, the increase in CD151 protein is potentially attributable to the simultaneous elevation in α3 integrin [18]. However, it is still unclear as to the mechanism of CD151 mRNA upregulation in glioblastomas. Our analyses of TCGA glioblastomas reveal minimal change in the copy number of CD151 or α3 integrin gene (data not shown), implicating a mechanism other than gene amplification or copy gain. Meanwhile, there is evidence that CD151 expression in gliomas is regulated by micro-RNAs or epigenetic regulation [34, 36]. In particular, mutation of IDH1 gene in gliomas has been shown to influence the methylation of a number of pro-malignant genes [3, 37], and is strongly associated with CD151 expression in our patient cohort (Fig. 1D). Thus, there is a likelihood that the aberrant expression of CD151 or α3β1 integrin in high-grade gliomas may result from epigenetic regulation.

### Functional contribution of CD151 and α3β1 integrin to glioblastoma malignancy

Although the RGD-based integrins, such as α5β1 integrin, are shown to promote survival and drug resistance of glioblastoma cells [12, 38], disruption of CD151 or α3β1 integrin appears to have minimal influence on the proliferation or survival of glioblastoma cells (data not shown). Rather, CD151 and α3β1 integrin drive the aggressiveness of glioblastoma by accelerating tumor cell motility and invasiveness (Figs. 2, 5, 6). These findings are also in line with recent functional studies of these molecules in glioblastomas [7–9, 39]. Given the high expression of multiple laminin isoforms in glioblastoma cells [8, 9, 40], CD151-α3β1 integrin complexes may act in an autocrine fashion. This notion is also supported by recent observations of α3β1 integrin function across multiple cancer types [41]. Moreover, because normal brain tissues are highly enriched in laminins [42], the pro-malignant roles of CD151-α3β1 integrin complexes in glioblastomas are likely strengthened by the tumor microenvironments.

Our data also implicate that the impact of CD151-α3β1 integrin complexes on the malignant behaviors of glioblastomas may occur in the context of TEMs. Notably, functional loss of tetraspanin CD9 led to a marked increase in EGF-stimulated tumor cell invasion, consistent with recent analyses of CD81 and its associated EWI-2 protein complexes in gliomas [25]. Intriguingly, CD151 knockdown in multiple glioblastoma lines had minimal effect on the surface expression of its associated partners, as determined by our FACS analyses (Supplementary Fig. S2). Perhaps, CD151 promotes the clustering of its associated integrin molecules on the cell surface [20, 28]. How CD151 and CD9 molecules within the same protein complexes or TEM confer opposing cellular or signaling functions, however, still remains a mystery.

Of note, CD151 may also drive glioblastoma malignancy by affecting the stemness property of glioma cells. This tetraspanin has recently been linked to the dynamics of tumor-initiating or progenitors cells [43, 44]. Also, CD151-associated α3 and α6 integrins are shown to enhance the aggressive behaviors of cancer stem cells [7, 10]. Thus, identification of a stem cell-related link will shed new light on the importance of CD151-LB integrin complexes or associated TEMs in glioblastoma malignancy.

### Signaling role of CD151-α3β1 integrin complexes in glioblastomas

The strong cooperation or synergy between CD151-LB integrins complexes and EGFR in glioblastomas revealed by our study (Figs. 3–6) is of high translational significance, as aberrant activation of this RTK occurs in >70% gliomas [4]. This type of signaling crosstalk is also consistent with our recent analyses of CD151 function in other malignancies, including breast and skin cancers [21, 28]. In addition, our observation of the activation of FAK as reflected by the enhanced phosphorylation of Y^397^ residue upon the engagement of laminin-5 and CD151-integrin complexes is in line with the pro-malignant role of this non-receptor tyrosine kinase in glioblastomas [45]. Beside the phosphorylation of Y^397^ residue, FAK may convey CD151-integrin complex-mediated signaling through the phosphorylation of Y^925^ residue upon EGF stimulation [46, 47]. Furthermore, we found that CD151-α3β1 integrin complexes signal through Graf (ARHGAP26)/small GTPase-dependent pathways in glioblastoma cells (Fig. 6). In contrast to a prior report on ELMO1, a regulator of GTPase activation and glioma cell motility [33], our study suggest that the crosstalk between the CD151-integrin complexes and RTKs converge at ARHGAP26, consistent with its link to the EGFR-mediated cell motility and signaling [48]. Collectively, our data suggest that CD151-α3β1 integrin complexes and EGFR converge at FAK and Graf (ARHGAR26) to drive the motility and invasiveness of glioblastoma.

The contribution of CD151 and associated LB integrins to glioma aggressiveness may not be completely dependent on their crosstalk with the EGFR pathway. In fact, CD151 and LB integrins have been shown to collaborate with multiple oncogenic RTKs (e.g., c-Met and ErbB2) or Ras/MAPK pathways [15, 28, 49] or Myc-driven network [50]. As these oncogenic events frequently occur in glioblastoma [26, 27, 51], the cooperation between CD151-α3β1 integrin complexes and EGFR detected in the current study may only reflect part of broad contributions of such complexes to the aggressiveness of such disease. However, it is unlikely that the signaling effect of CD151–integrin complex detected in the current study is attributed to their impact on the EGFR activation, particularly its autophosphorylation, according to our recent studies [15, 52].

It is worth noting that the pro-malignant function of CD151-integrin complexes in glioblastoma occurs in the context of cell-ECM interaction, consistent with prior studies in other cancer types [52, 53]. However, such adhesion molecules are also capable of suppressing tumorigenesis, particularly in the presence of intact cell-cell contact or E-cadherin [54, 55].

### Implications of CD151-α3β1 integrin complexes and associated pathways as therapeutic targets

Results from the present study strongly support the prognostic and therapeutic potential of CD151-α3β1 integrin complexes and their associated pathways for glioblastoma. Experimentally, the pro-malignant roles of CD151 or α3β1 integrin are readily disrupted by administration of function-blocking monoclonal antibodies, such as 1A5 or Tsr151 for CD151, and IIF5 or P1B5 for α3 integrin [21]. The functional disruption of these molecules is also achievable through inhibition of downstream signaling, such as FAK activation (Fig. 5). Given the emerging link of these molecules to the stemness of glioblastoma [7, 10], targeting CD151-LB integrin complexes may also provide an effective means against tumor recurrence.

In summary, our study demonstrates that CD151 and its associated α3 integrin are markedly elevated in malignant gliomas, and synergize with EGFR-dependent signaling pathways to drive the aggressive behaviors of glioblastoma cells. As such, our findings provide crucial evidence of CD151-integrin complexes and associated pathways as prognostic markers and therapeutic targets for glioblastoma or other aggressive gliomas.

## MATERIALS AND METHODS

### Antibodies, reagents, and cell cultures

CD151 monoclonal antibodies include 5C11, 1A5 and FITC-conjugated IIG5a (GeneTex, San Antonia, TX). Anti-CD9 monoclonal antibody MM2/57 (unconjugated and FITC conjugated) was from Biosource (San Diego, CA). The monoclonal antibodies to CD81 (M38) or integrin α2 (IIE10), α3 (X8), α6 (GÖH3), β1 (TS2/16), and β4 (3E1) were described elsewhere [15, 16]. Antibodies to FAK and Y^397^-phosphorylated FAK were obtained from Santa Cruz Biotechnology (Santa Cruz, CA). Antibodies to small GTPases (Rac1 and Cdc42) were obtained from Cell Signaling Technology (Danvers, MA). Small molecule-based inhibitors against FAK (TAE226) or EGFR/ErbB2 (lapatinib or gefitinib) were described in a prior study [15].

Antibodies used for IHC analyses of CD151 and α3 integrin were obtained from Leica Microsystems (Lawrenceville, GA) or prepared in-house [54]. *IDH1* mutation status was examined by using an anti-R132H IDH1 antibody (HistoBioTec, Miami Beach, FL). Human glioblastoma cell lines LN827, LN428 and LN308 were obtained from Dr. Erwin Van Meir (Emory University, Atlanta, GA) [56]. LN229, LN373, and U87 lines were purchased from ATCC (Manassas, VA). For xenograft analysis, LN229 cells were engineered to express luciferase as previously described [57]. No authentication was conducted on all cell lines used in the current study. All cell lines were cultured in DMEM or RPMI-1640 supplemented with 10% fetal bovine serum, 10 mmol/L HEPES, and antibiotics (Invitrogen, Carlsbad, CA). All cultures were maintained at 37°C in a humidified atmosphere of 5% CO_2_. EGF was obtained from UBI (Lake Placid, NY). Matrigel and fibronectin were obtained from BD Biosciences (San Jose, CA). Laminin-5 was prepared for the condition medium of A431 carcinoma cells as previously described [30].

### Transient and stable knockdown of tetraspanins and integrins using siRNA and shRNA

For all transient knockdowns, on-target siRNAs were purchased from Dharmacon (Lafayette, CO) and delivered to tumor cells using Lipofectamine-2000 (Invitrogen). For stable knockdown, human CD151 or β1 integrin oligos were cloned into lentivirus expression vector plenti-U6BX and verified by DNA sequencing and mutagenesis analyses as previously described [28]. The infected cells were sorted on flow cytometry by double sorting with GFP and with specific monoclonal antibodies for selection of stable CD151 or β1 integrin knockdown cell lines.

### Cell sorting and FACS analyses

Cell sorting was carried out to obtain glioblastoma cell lines infected with lentivirus-expressing control or CD151-specific shRNA as previously described [28]. Virus-infected tumor cells were initially stained with anti-CD151 monoclonal antibodies (5C11) and APC-conjugated secondary antibodies on ice, followed by two-way sorting on flow cytometry at our local core facility. For FACS analyses of the expression of cell surface molecules, tumor cells were stained with the indicated primary monoclonal antibodies, followed by fluorescence-conjugated secondary antibodies prior to analyses on flow cytometry. For each FACS analyses, a total of 5–10 × 10^3^ cells were analyzed and mean fluorescence intensity values were calculated.

### Tumor cell motility, invasion, and proliferation assays

Analyses of random cell motility were conducted by imaging live tumor cells at 20 min intervals for 12 h with a Nikon Automated Eclipse Ti-E inverted microscope and an OKALAB incubator equipped with a heater and gas mixer constant at 37°C and 5% CO_2_. Nikon NIS-Elements Advanced Analysis Software was employed to estimate distance traveled by tumor cells as described in our prior study [15]. For analyses of tumor cell invasion, cells were detached using non-enzymatic EDTA-containing dissociation buffer (Life Technologies, Grand Island, NY). Cells were then suspended in serum-free DMEM with 0.02% bovine serum albumin, and added to trans-well chambers containing 8-μm membranes pre-coated with Matrigel (BD Biosciences). Chamber bottoms contained serum-free medium with or without presence of 10 ng/mL EGF. After invasion through Matrigel (12–18 h, 37°C), membranes were washed, dried, fixed, and stained with Giemsa (Sigma-Aldrich, St. Louis, MO). Proliferation of human glioblastoma cells was evaluated by MTT assay. Fold changes were calculated relative to the control without the presence of a stimulant.

### Immunoprecipitation, [^3^H]-palmitate labeling, and signaling assays, and immunoblot analyses

For metabolic labeling, siRNA-treated cells (80–90% confluent) were washed in PBS, serum starved (3–4 h), pulsed for 1–2 h in medium containing 0.2 to 0.3 mCi/mL [^3^H]-palmitic acid plus 5% dialyzed FBS, and then lysed in 1% Brij-96 for 5 h at 4°C. Immunoprecipitation and detection of [^3^H]-palmitate-labeled proteins were completed as previously described (12, 30, 31). To assess protein phosphorylation, cells were lysed in RIPA buffer containing 1% Triton X-100, 1% deoxycholate, and 0.1% SDS.

For signaling analyses, cells were seeded onto 6-well plates (2 × 10^5^ cells per well) and starved overnight prior to stimulation. Cells were lysed in RIPA buffer supplemented with 1 mM Na_3_VO_4,_ PMSF and protease inhibitor cocktail [30]. Protein concentrations were quantified by use of DC Protein Assay Kit (Bio-Rad, Hercules, CA). Then phosphorylated proteins were either immunoprecipitated and blotted with anti-phosphotyrosine antibody (4G10, UBI) or directly blotted in cell lysates using phospho-specific antibodies. Rac1 activation was assessed with a glutathione S-transferase (GST)-PBD pull-down assay kit (UBI).

Immunoblotting or Western analyses were conducted by separating equal amounts of proteins on SDS-PAGE and transferring samples onto a nitrocellulose membrane prior to blotting detection with the indicated antibodies. For evaluation of the differences in protein expression, ratios of densitometry values of individual protein to β-actin were calculated. These values were subsequently divided by the smallest value within the group to obtain a fold change.

### Tissue microarrays of glioblastomas and IHC analyses

A set of tissue microarrays (TMAs) were prepared in-house using archived formalin-fixed, paraffin-embedded gliomas as previously described [45]. A total of 96 cases comprised the TMAs, including 9 WHO grade II astrocytomas, 11 grade III astrocytomas, 12 anaplastic grade III oligodendrogliomas, 16 grade II oligodendrogliomas, and 47 grade IV glioblastomas. A commercial TMA of glioblastomas was also purchased from US Biomax (Rockville, MD). IHC staining of paraffin-embedded TMAs was performed as previously described [45]. In brief, secondary antibodies were EnVision labeled polymer-HRP (horseradish peroxidase) anti-mouse or anti-rabbit as appropriate. Staining was visualized using 3, 3′-diaminobenzidine (DAB) chromogen (Dako, Copenhagen, CA). The TMAs stained with antibodies against CD151 were digitally quantified via ScanScope XT whole slide scanner, followed by analysis with Aperio Spectrum Version 11.2.0.780 software. For technical reasons, the TMA cores stained with α3 integrin were difficult to reliably score digitally, so instead they were visually semi-quantified by C.H. on a relative scale from 0 to 3 (0 = negative, 1 = weak, 2 = intermediate, 3 = strong).

For both digital and visual scoring, results from all 3 cores were averaged together to produce a final score for a tumor. The scoring of immunostaining was evaluated on the basis of staining intensity and percentages of positively stained areas.

### Tumor xenograft analyses

All animal experiments were carried out in accordance with protocols approved by the Institutional Animal Care and Use Committee at the Dana-Farber Cancer Institute (Boston, MA). Control or CD151 knockdown LN229 were infected with luciferase-expressing retrovirus, selected with G418 and subsequently verified for the expression of luciferase or CD151 by immunoblotting. For orthotopic analyses, the luciferase-expressing tumor cells were dissociated and resuspended in HBSS at a density of 1.0 × 10^5^ cells/μL. A total of 2.0 μL of tumor cell mixture was injected into the striatum of immunocompromised nude mice in the position of 2 mm posterior to the bregma, 2 mm laterally, and 2 mm deep to the dura, as described in our prior study [57]. Changes in tumor sizes were determined on the basis of chemoluminescence intensity under a Xenogen imager. In the course of the study, all animals were euthanized at the onset of neurological symptoms or once moribund. For *in vivo* imaging, mice were anesthetized, given D-luciferin at 50 mg/mL by intraperitoneal (I.P.) injection (Xenogen, Alameda, CA), and imaged with the IVIS Imaging System (Xenogen) for 10–120 s, bin size 2. Data were normalized to bioluminescence at the initiation of treatment for each animal.

### Statistical analysis

All experiments described in the study were independently repeated. Differences between groups were analyzed via two-tailed *t*-test or one-way ANOVA with post-hoc Dunn's test, as appropriate. Correlation between CD151 and α3 integrin expression was measured via linear regression. Survival differences were compared via log-rank (Mantel-Cox) test. Multivariate survival analysis was done via Cox proportional hazards survival regression. Statistical significance was reached when *p* < 0.05. All analyses were done via GraphPad software (La Jolla, CA), except for the Cox proportional hazards survival regression which was done using http://statpages.org/.

## SUPPLEMENTARY FIGURES



## References

[1] Weller M, Pfister SM, Wick W, Hegi ME, Reifenberger G, Stupp R (2013). Molecular neuro-oncology in clinical practice: a new horizon. The lancet oncology.

[2] Vehlow A, Cordes N (2013). Invasion as target for therapy of glioblastoma multiforme. Biochimica et biophysica acta.

[3] Dunn GP, Rinne ML, Wykosky J, Genovese G, Quayle SN, Dunn IF, Agarwalla PK, Chheda MG, Campos B, Wang A, Brennan C, Ligon KL, Furnari F, Cavenee WK, Depinho RA, Chin L (2012). Emerging insights into the molecular and cellular basis of glioblastoma. Genes & development.

[4] Francis JM, Zhang CZ, Maire CL, Jung J, Manzo VE, Adalsteinsson VA, Homer H, Haidar S, Blumenstiel B, Pedamallu CS, Ligon AH, Love JC, Meyerson M, Ligon KL (2014). EGFR variant heterogeneity in glioblastoma resolved through single-nucleus sequencing. Cancer discovery.

[5] Gini B, Mischel PS (2014). Greater than the sum of its parts: single-nucleus sequencing identifies convergent evolution of independent EGFR mutants in GBM. Cancer discovery.

[6] Hynes RO (2002). Integrins: Bidirectional, allosteric signaling machines. Cell.

[7] Nakada M, Nambu E, Furuyama N, Yoshida Y, Takino T, Hayashi Y, Sato H, Sai Y, Tsuji T, Miyamoto KI, Hirao A, Hamada JI (2013). Integrin alpha3 is overexpressed in glioma stem-like cells and promotes invasion. British journal of cancer.

[8] Fukushima Y, Ohnishi T, Arita N, Hayakawa T, Sekiguchi K (1998). Integrin alpha 3 beta 1-mediated interaction with laminin-5 stimulates adhesion, migration and invasion of malignant glioma cells. International Journal of Cancer.

[9] Kawataki T, Yamane T, Naganuma H, Rousselle P, Anduren I, Tryggvason K, Patarroyo M (2007). Laminin isoforms and their integrin receptors in glioma cell migration and invasiveness: Evidence for a role of alpha 5-laminin(s) and alpha 3 beta 1 integrin. Experimental cell research.

[10] Lathia JD, Gallagher J, Heddleston JM, Wang J, Eyler CE, Macswords J, Wu Q, Vasanji A, McLendon RE, Hjelmeland AB, Rich JN (2010). Integrin alpha 6 regulates glioblastoma stem cells. Cell stem cell.

[11] Stipp CS (2010). Laminin-binding integrins and their tetraspanin partners as potential antimetastatic targets. Expert Reviews in Molecular Medicine.

[12] Janouskova H, Maglott A, Leger DY, Bossert C, Noulet F, Guerin E, Guenot D, Pinel S, Chastagner P, Plenat F, Entz-Werle N, Lehmann-Che J, Godet J, Martin S, Teisinger J, Dontenwill M (2012). Integrin alpha5beta1 plays a critical role in resistance to temozolomide by interfering with the p53 pathway in high-grade glioma. Cancer Res.

[13] Tabatabai G, Tonn JC, Stupp R, Weller M (2011). The role of integrins in glioma biology and anti-glioma therapies. Current pharmaceutical design.

[14] Hemler ME (2008). Targeting of tetraspanin proteins - potential benefits and strategies. Nature Reviews Drug Discovery.

[15] Deng X, Li Q, Hoff J, Novak M, Yang H, Jin H, Erfani SF, Sharma C, Zhou P, Rabinovitz I, Sonnenberg A, Yi Y, Stipp CS, Kaetzel DM, Hemler ME, Yang XH (2012). Integrin-Associated CD151 Drives ErbB2-Evoked Mammary Tumor Onset and Metastasis. Neoplasia.

[16] Yang X, Kovalenko OV, Tang W, Claas C, Stipp CS, Hemler ME (2004). Palmitoylation supports assembly and function of integrin-tetraspanin complexes. Journal of Cell Biology.

[17] Hemler ME (2005). Tetraspanin functions and associated microdomains. Nature Reviews Molecular Cell Biology.

[18] Kazarov AR, Yang XW, Stipp CS, Sehgal B, Hemler ME (2002). An extracellular site on tetraspanin CD151 determines alpha 3 and alpha 6 integrin-dependent cellular morphology. Journal of Cell Biology.

[19] Rubinstein E (2011). The complexity of tetraspanins. Biochem Soc Trans.

[20] Yang XH, Mirchev R, Deng X, Yacono P, Yang HL, Golan DE, Hemler ME (2012). CD151 restricts alpha6 integrin diffusion mode. J Cell Sci.

[21] Hemler ME (2014). Tetraspanin proteins promote multiple cancer stages. Nature reviews Cancer.

[22] Romanska HM, Berditchevski F (2011). Tetraspanins in human epithelial malignancies. Journal of Pathology.

[23] Budczies J, Klauschen F, Sinn BV, Gyorffy B, Schmitt WD, Darb-Esfahani S, Denkert C (2012). Cutoff Finder: a comprehensive and straightforward Web application enabling rapid biomarker cutoff optimization. PloS one.

[24] Horbinski C (2013). What do we know about IDH1/2 mutations so far, and how do we use it?. Acta neuropathologica.

[25] Kolesnikova TV, Kazarov AR, Lemieux ME, Lafleur MA, Kesari S, Kung AL, Hemler ME (2009). Glioblastoma inhibition by cell surface immunoglobulin protein EWI-2, *in vitro* and *in vivo*. Neoplasia.

[26] Haynes HR, Camelo-Piragua S, Kurian KM (2014). Prognostic and predictive biomarkers in adult and pediatric gliomas: toward personalized treatment. Frontiers in oncology.

[27] Vitucci M, Hayes DN, Miller CR (2011). Gene expression profiling of gliomas: merging genomic and histopathological classification for personalised therapy. British journal of cancer.

[28] Yang XWH, Richardson AL, Torres-Arzayus MI, Zhou PC, Sharma C, Kazarov AR, Andzelm MM, Strominger JL, Brown M, Hemler ME (2008). CD151 accelerates breast cancer by regulating alpha(6) integrin function, signaling, and molecular organization. Cancer Res.

[29] Yang X, Claas C, Kraeft SK, Chen LB, Wang Z, Kreidberg JA, Hemler ME (2002). Palmitoylation of tetraspanin proteins: modulation of CD151 lateral interactions, subcellular distribution, and integrin-dependent cell morphology. Molecular biology of the cell.

[30] Yang XH, Flores LM, Li Q, Zhou P, Xu F, Krop IE, Hemler ME (2010). Disruption of laminin-integrin-CD151-focal adhesion kinase axis sensitizes breast cancer cells to ErbB2 antagonists. Cancer Res.

[31] Fortin Ensign SP, Mathews IT, Symons MH, Berens ME, Tran NL (2013). Implications of Rho GTPase Signaling in Glioma Cell Invasion and Tumor Progression. Frontiers in oncology.

[32] Choma DP, Pumiglia K, DiPersio CM (2004). Integrin alpha 3 beta 1 directs the stabilization of a polarized lamellipodium in epithelial cells through activation of Rac1. J Cell Sci.

[33] Jarzynka MJ, Hu B, Hui KM, Bar-Joseph I, Gu W, Hirose T, Haney LB, Ravichandran KS, Nishikawa R, Cheng SY (2007). ELMO1 and Dock180, a bipartite Rac1 guanine nucleotide exchange factor, promote human glioma cell invasion. Cancer Res.

[34] Lee D, Suh YL, Park TI, Do IG, Seol HJ, Nam DH, Kim ST (2013). Prognostic significance of tetraspanin CD151 in newly diagnosed glioblastomas. Journal of surgical oncology.

[35] Ha SY, Kang SY, Do IG, Suh YL (2013). Glioblastoma with oligodendroglial component represents a subgroup of glioblastoma with high prevalence of IDH1 mutation and association with younger age. Journal of neuro-oncology.

[36] Han ZB, Yang Z, Chi Y, Zhang L, Wang Y, Ji Y, Wang J, Zhao H, Han ZC (2013). MicroRNA-124 suppresses breast cancer cell growth and motility by targeting CD151. Cellular physiology and biochemistry : international journal of experimental cellular physiology, biochemistry, and pharmacology.

[37] Sturm D, Bender S, Jones DT, Lichter P, Grill J, Becher O, Hawkins C, Majewski J, Jones C, Costello JF, Iavarone A, Aldape K, Brennan CW, Jabado N, Pfister SM (2014). Paediatric and adult glioblastoma: multiform (epi)genomic culprits emerge. Nature reviews Cancer.

[38] Martin S, Janouskova H, Dontenwill M (2012). Integrins and p53 pathways in glioblastoma resistance to temozolomide. Frontiers in oncology.

[39] Kohno M, Hasegawa H, Miyake M, Yamamoto T, Fujita S (2002). CD151 enhances cell motility and metastasis of cancer cells in the presence of focal adhesion kinase. International Journal of Cancer.

[40] Ljubimova JY, Lakhter AJ, Loksh A, Yong WH, Riedinger MS, Miner JH, Sorokin LM, Ljubimov AV, Black KL (2001). Overexpression of alpha 4 chain-containing laminins in human glial tumors identified by gene microarray analysis. Cancer Res.

[41] Zhou B, Gibson-Corley KN, Herndon ME, Sun Y, Gustafson-Wagner E, Teoh-Fitzgerald M, Domann FE, Henry MD, Stipp CS (2014). Integrin alpha3beta1 can function to promote spontaneous metastasis and lung colonization of invasive breast carcinoma. Molecular cancer research: MCR.

[42] Durbeej M (2010). Laminins. Cell and tissue research.

[43] Rajasekhar VK, Studer L, Gerald W, Socci ND, Scher HI (2011). Tumour-initiating stem-like cells in human prostate cancer exhibit increased NF-kappaB signalling. Nat Commun.

[44] Yin Y, Deng X, Liu Z, Baldwin LA, Lefringhouse J, Zhang J, Hoff JT, Erfani SF, Rucker EB, O'Connor K, Liu C, Wu Y, Zhou BP, Yang XH (2014). CD151 represses mammary gland development by maintaining the niches of progenitor cells. Cell Cycle.

[45] Natarajan M, Stewart JE, Golemis EA, Pugacheva EN, Alexandropoulos K, Cox BD, Wang W, Grammer JR, Gladson CL (2006). HEF1 is a necessary and specific downstream effector of FAK that promotes the migration of glioblastoma cells. Oncogene.

[46] Sieg DJ, Hauck CR, Ilic D, Klingbeil CK, Schaefer E, Damsky CH, Schlaepfer DD (2000). FAK integrates growth-factor and integrin signals to promote cell migration. Nature cell biology.

[47] Flinder LI, Timofeeva OA, Rosseland CM, Wierod L, Huitfeldt HS, Skarpen E (2011). EGF-induced ERK-activation downstream of FAK requires rac1-NADPH oxidase. Journal of cellular physiology.

[48] Simpson KJ, Selfors LM, Bui J, Reynolds A, Leake D, Khvorova A, Brugge JS (2008). Identification of genes that regulate epithelial cell migration using an siRNA screening approach. Nature cell biology.

[49] Franco M, Muratori C, Corso S, Tenaglia E, Bertotti A, Capparuccia L, Trusolino L, Comoglio PM, Tamagnone L (2010). The tetraspanin CD151 is required for Met-dependent signaling and tumor cell growth. J Biol Chem.

[50] Bredel M, Bredel C, Juric D, Harsh GR, Vogel H, Recht LD, Sikic BI (2005). Functional network analysis reveals extended gliomagenesis pathway maps and three novel MYC-interacting genes in human gliomas. Cancer Res.

[51] Chen J, McKay RM, Parada LF (2012). Malignant glioma: lessons from genomics, mouse models, and stem cells. Cell.

[52] Yang XH, Richardson AL, Torres-Arzayus MI, Zhou P, Sharma C, Kazarov AR, Andzelm MM, Strominger JL, Brown M, Hemler ME (2008). CD151 accelerates breast cancer by regulating alpha 6 integrin function, signaling, and molecular organization. Cancer Res.

[53] Zijlstra A, Lewis J, Degryse B, Stuhlmann H, Quigley JP (2008). The inhibition of tumor cell intravasation and subsequent metastasis via regulation of *in vivo* tumor cell motility by the tetraspanin CD151. Cancer cell.

[54] Baldwin LA, Hoff JT, Lefringhouse J, Zhang M, Jia C, Liu Z, Erfani S, Jin H, Xu M, She QB, van Nagell JR, Wang C, Chen L, Plattner R, Kaetzel DM, Luo J (2014). CD151-alpha3beta1 integrin complexes suppress ovarian tumor growth by repressing slug-mediated EMT and canonical Wnt signaling. Oncotarget.

[55] Varzavand A, Drake JM, Svensson RU, Herndon ME, Zhou B, Henry MD, Stipp CS (2013). Integrin alpha3beta1 regulates tumor cell responses to stromal cells and can function to suppress prostate cancer metastatic colonization. Clinical & experimental metastasis.

[56] Redjal N, Chan JA, Segal RA, Kung AL (2006). CXCR4 inhibition synergizes with cytotoxic chemotherapy in gliomas. Clinical cancer research : an official journal of the American Association for Cancer Research.

[57] Rubin JB, Kung AL, Klein RS, Chan JA, Sun Y, Schmidt K, Kieran MW, Luster AD, Segal RA (2003). A small-molecule antagonist of CXCR4 inhibits intracranial growth of primary brain tumors. Proceedings of the National Academy of Sciences of the United States of America.

