# Supplementary material for: CD151-α3β1 integrin complexes are prognostic markers of glioblastoma and cooperate with EGFR to drive tumor cell motility and invasion
Source: Oncotarget. 2015 Aug 13;6(30):29675–93. doi: 10.18632/oncotarget.4896 (PMC4745755; doi:10.18632/oncotarget.4896)
Supplement: Supplementary file 1 [file oncotarget-06-29675-s001.pdf]

SUPPLEMENTARY FIGURES

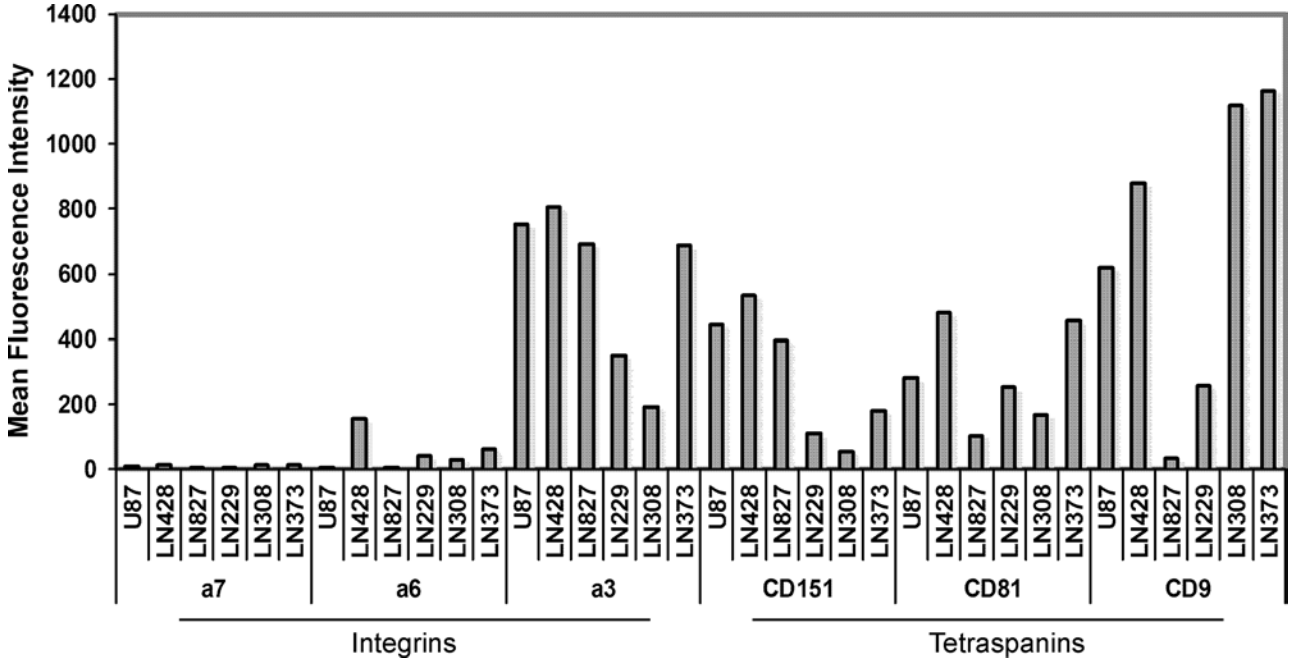

Supplementary Figure S1: Screening of surface expression of tetraspanins and LB integrins in human glioblastoma cell lines. Glioblastoma cell lines (U87, LN428, LN8297, LN229, LN308, and LN373) were stained with monoclonal antibodies against α7 (8G2), α6 (GöH3), α3 (X8), CD151 (5C11), CD81 (M38), and CD9 (MM2/F7), followed by staining with FITC-conjugated secondary antibody and analyses on flow cytometry. Values: mean florescence intensity (MFI).

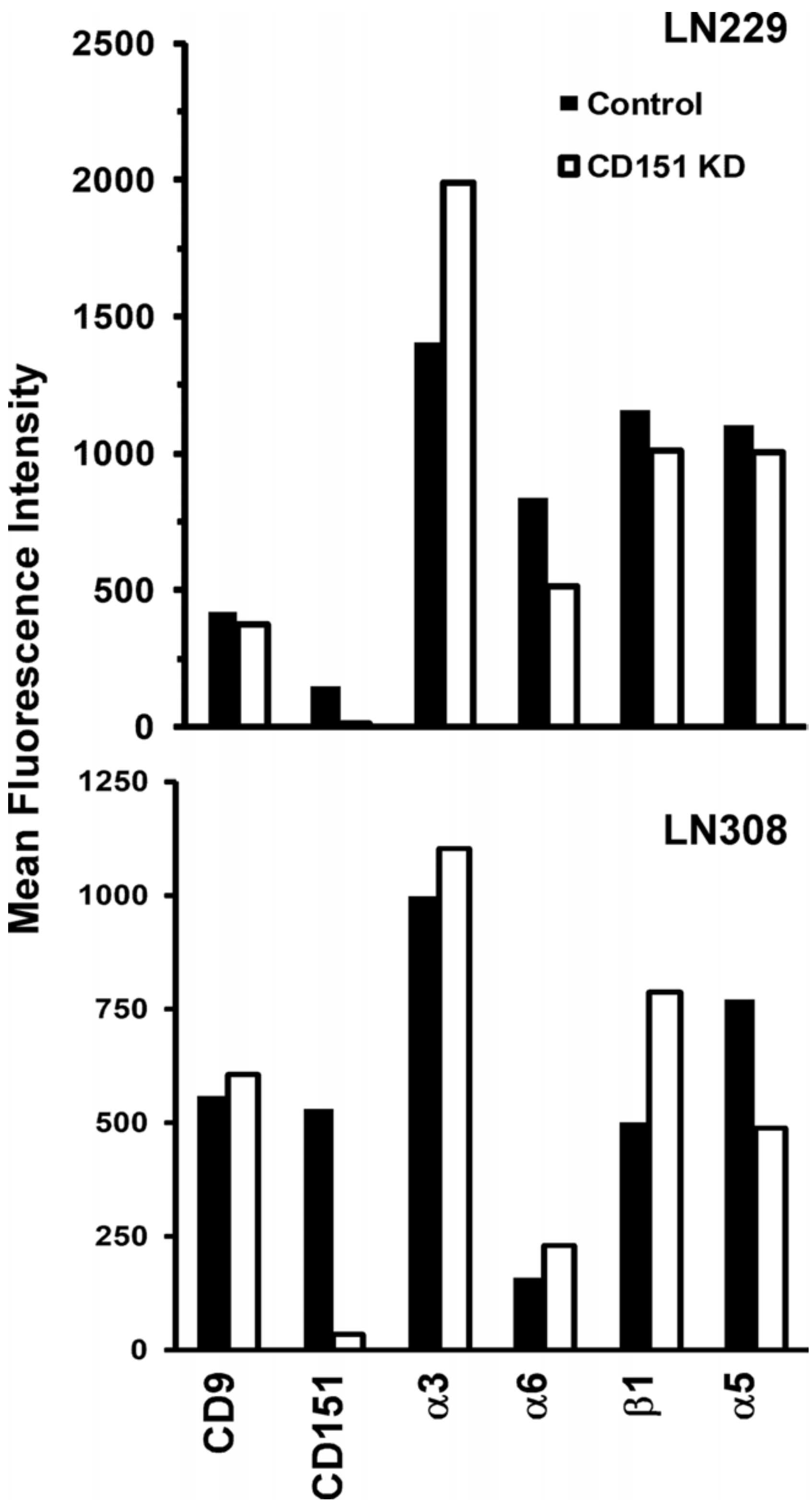

Supplementary Figure S2: The effect of CD151 knockdown on the surface expression of CD151-associated integrins and tetraspanins in glioblastoma cells. LN229 and LN308 cells with control and stable CD151 knockdown were analyzed by flow cytometry with the indicated monoclonal antibodies. Values: mean florescence intensity (MFI).

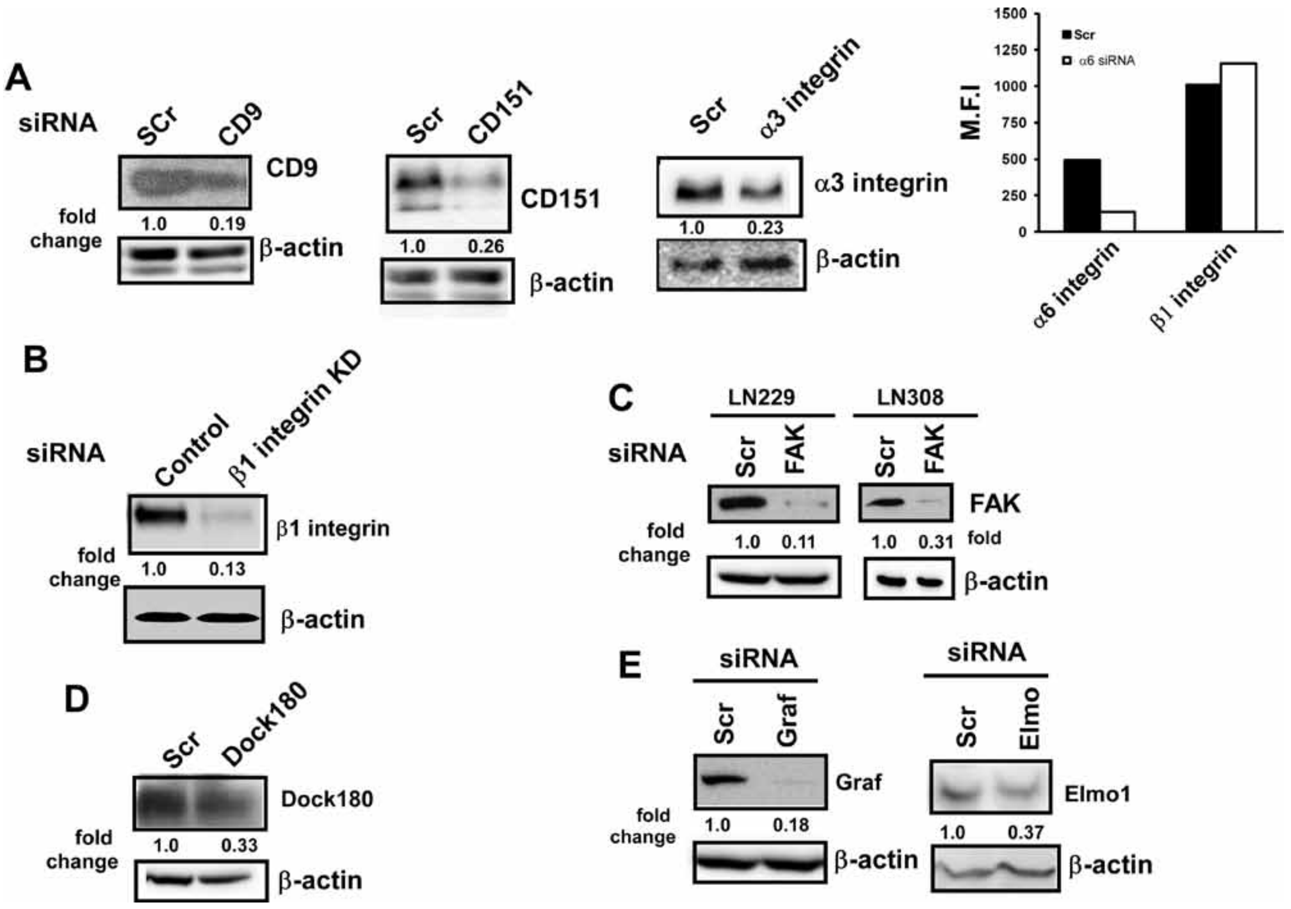

Supplementary Figure S3: Evaluation of the efficiency of siRNA- or shRNA-based knockdown of CD151 and associated molecules. Tumor cells were lysed in RIPA buffer for subsequent immunoblotting or detached via non-enzymatic buffer and subsequently analyzed for the surface expression of the indicated molecules.
